# Supplementary material for: Functional Outcomes Following Hip Replacement in Community-Dwelling Older Adults
Source: J Clin Med. 2022 Aug 30;11(17):5117. doi: 10.3390/jcm11175117 (PMC9457152; doi:10.3390/jcm11175117)
Supplement: Supplementary file 1 [file jcm-11-05117-s001.zip › jcm-1816104-supplementary.pdf]

Figure S1. Baseline and follow-up measures of self-reported physical and mental health function in participants with hip replacement and age- and sex-matched controls, data presented as mean (standard deviation)

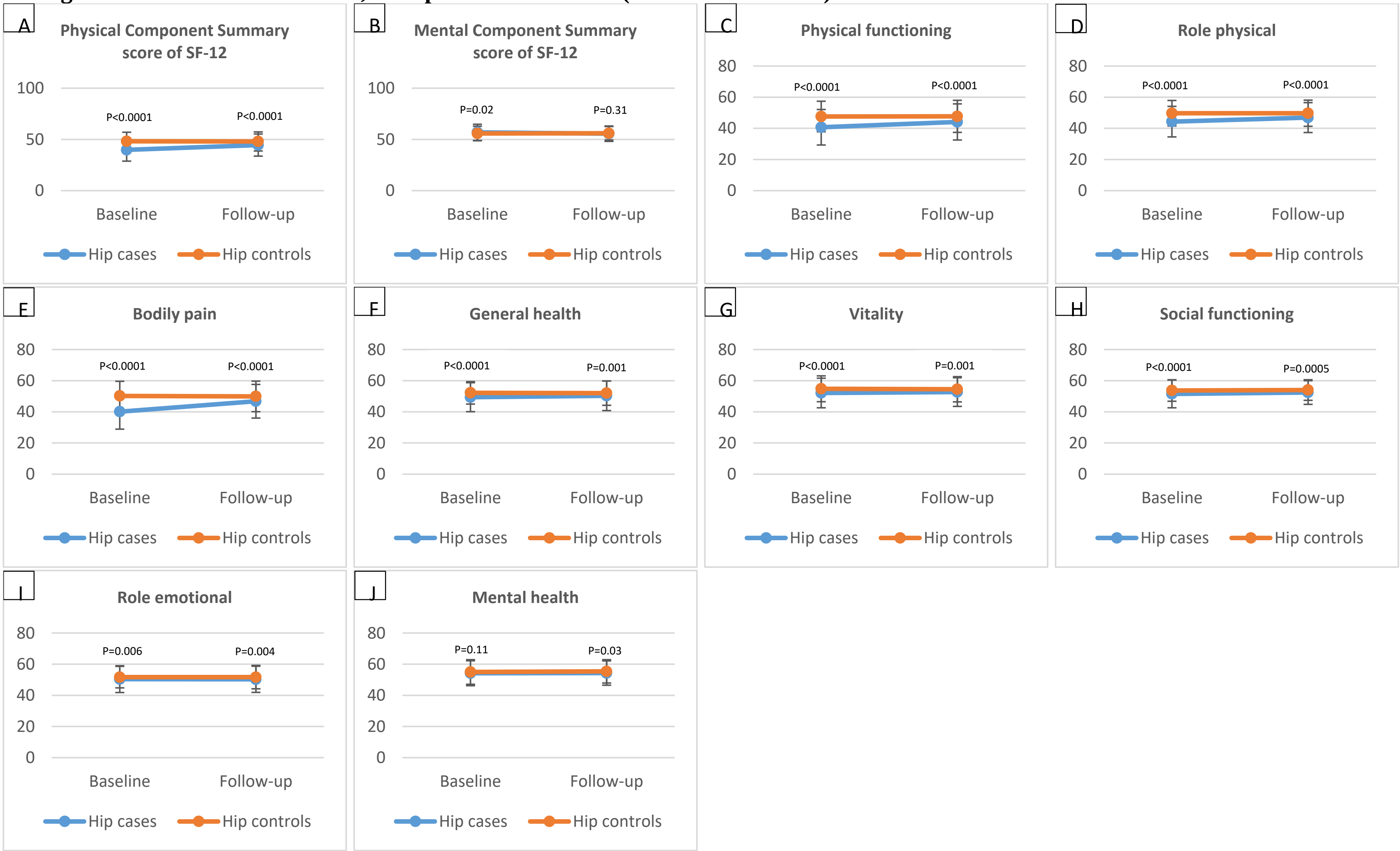

Table S1. Baseline and follow-up measures of health status and gait speed by gender in participants with hip replacement and age- and sex-matched controls

	Participants with hip replacement			Age- and sex-matched controls			Difference between groups		
	Baseline (preoperative) Mean (SD)	Follow-up Mean (SD)	Change Mean (SD)	Baseline (study entry) Mean (SD)	Follow-up Mean (SD)	Change Mean (SD)	Baseline P[c]	Follow-up P[c]	Change P[c]
**Females**	*n=260*			*n=363*					
PCS	37.9 (11.1)	42.9 (11.4)	5.0 (10.7)	47.5 (9.1)	46.7 (9.9)	-0.8 (7.3)	<0.0001	<0.0001	<0.0001
MCS	56.9 (8.1)	55.4 (7.9)	-1.5 (8.7)	55.3 (7.6)	55.8 (7.1)	0.5 (7.7)	0.01	0.55	0.003
Physical function	38.2 (11.3)	41.2 (12.0)	3.0 (11.2)	46.0 (10.1)	45.5 (10.8)	-0.5 (8.7)	<0.0001	<0.0001	<0.0001
Role physical	42.8 (9.9)	46.4 (9.7)	3.6 (10.2)	49.1 (8.6)	49.3 (8.5)	0.2 (8.2)	<0.0001	0.0001	<0.0001
Bodily pain	38.7 (11.5)	45.9 (11.3)	7.1 (12.5)	49.5 (9.7)	48.6 (10.6)	-0.9 (9.7)	<0.0001	0.002	<0.0001
General health	49.0 (9.7)	49.7 (10.5)	0.7 (10.0)	52.3 (7.0)	51.8 (7.7)	-0.6 (6.9)	<0.0001	0.004	0.07
Vitality	51.2 (9.3)	51.9 (9.3)	0.7 (9.2)	54.6 (8.5)	54.2 (8.5)	-0.4 (7.8)	<0.0001	0.001	0.11
Social functioning	50.6 (9.5)	51.7 (8.3)	1.0 (9.7)	53.5 (7.1)	53.5 (7.0)	-0.03 (7.6)	<0.0001	0.004	0.12
Role emotional	50.0 (9.0)	49.5 (9.1)	-0.6 (10.1)	50.9 (7.6)	51.2 (7.7)	0.3 (8.2)	0.18	0.01	0.26
Mental health	53.5 (8.0)	53.9 (8.0)	0.4 (8.1)	53.7 (8.1)	54.3 (7.7)	0.6 (8.4)	0.78	0.50	0.71
Gait speed (m/sec)[a]	0.92 (0.26)	0.89 (0.21)	-0.02 (0.23)	1.03 (0.22)	0.98 (0.23)	-0.05 (0.18)	<0.0001	<0.0001	0.14
**Males**	*n=185*			*n=265*					
PCS	42.5 (10.3)	46.8 (9.4)	4.4 (10.2)	49.1 (8.3)	49.8 (8.1)	0.6 (6.8)	<0.0001	0.0005	<0.0001
MCS	57.0 (7.6)	55.7 (6.9)	-1.3 (7.5)	56.6 (6.3)	56.3 (5.9)	-0.3 (7.6)	0.53	0.36	0.18
Physical function	44.1 (10.7)	48.2 (9.8)	4.1 (12.0)	49.8 (8.9)	50.7 (8.7)	0.9 (8.2)	<0.0001	0.005	0.001
Role physical	46.4 (9.3)	47.6 (9.4)	1.2 (10.8)	50.7 (7.5)	50.4 (8.3)	-0.3 (7.2)	<0.0001	0.001	0.09
Bodily pain	42.1 (10.4)	48.2 (10.0)	6.1 (11.3)	51.2 (8.9)	51.8 (8.4)	0.6 (9.0)	<0.0001	<0.0001	<0.0001
General health	50.1 (8.8)	51.2 (7.8)	1.1 (8.6)	52.0 (7.4)	52.4 (7.9)	0.4 (7.2)	0.01	0.13	0.32
Vitality	53.4 (9.7)	53.8 (8.8)	0.5 (10.1)	55.2 (8.0)	54.9 (7.7)	-0.3 (8.5)	0.03	0.18	0.34
Social functioning	52.7 (7.9)	53.5 (6.4)	0.8 (8.7)	54.0 (6.7)	54.7 (5.9)	0.7 (7.4)	0.08	0.05	0.92
Role emotional	51.0 (7.9)	51.5 (7.2)	0.5 (8.4)	52.8 (5.7)	52.4 (7.2)	-0.4 (7.8)	0.005	0.18	0.25
Mental health	55.2 (7.9)	55.0 (7.5)	-0.2 (8.1)	56.8 (7.4)	56.7 (6.9)	-0.02 (8.8)	0.03	0.01	0.80
Gait speed (m/sec)[b]	1.02 (0.23)	1.02 (0.21)	-0.002 (0.22)	1.09 (0.23)	1.05 (0.21)	-0.04 (0.19)	0.005	0.19	0.06

SD: standard deviation; PCS: Physical component summary score of SF-12; MCS: Mental component summary score of SF-12
[a]n=190 for female participants with hip replacement and n=329 for female controls
[b]n=141 for male participants with hip replacement and n=245 for male controls
[c]For difference between participants with hip replacement and controls in each of the baseline and follow-up measures and their change from baseline to follow-up

Table S2. Changes in health status and gait speed from baseline to follow-up by gender in participants with hip replacement and age- and sex-matched controls

	Participants with hip replacement		Age- and sex-matched controls		Difference between groups	
	Mean change (95% CI)	P	Mean change (95% CI)	P	Mean difference (95% CI)	P
**Females**	*n=256*		*n=361*			
PCS	5.3 (4.1, 6.5)	<0.001	-0.6 (-1.6, 0.4)	0.24	5.9 (4.4, 7.4)	<0.001
MCS	-1.7 (-2.8, -0.6)	0.002	0.4 (-0.5, 1.3)	0.40	-2.1 (-3.5, -0.7)	0.004
Physical function	3.6 (2.3, 4.9)	<0.001	-0.5 (-1.5, 0.6)	0.41	4.1 (2.4, 5.8)	<0.001
Role physical	3.6 (2.4, 4.8)	<0.001	0.4 (-0.6, 1.3)	0.48	3.2 (1.7, 4.8)	<0.001
Bodily pain	7.2 (5.8, 8.7)	<0.001	-0.7 (-1.9, 0.6)	0.29	7.9 (6.0, 9.8)	<0.001
General health	0.5 (-0.6, 1.6)	0.36	-0.5 (-1.5, 0.4)	0.26	1.0 (-0.4, 2.5)	0.15
Vitality	0.8 (-0.3, 1.9)	0.14	-0.4 (-1.3, 0.6)	0.43	1.2 (-0.3, 2.6)	0.11
Social functioning	0.8 (-0.3, 1.8)	0.15	-0.2 (-1.1, 0.7)	0.69	1.0 (-0.4, 2.4)	0.18
Role emotional	-0.5 (-1.7, 0.7)	0.41	0.2 (-0.8, 1.2)	0.74	-0.7 (-2.2, 0.9)	0.40
Mental health	0.2 (-0.8, 1.3)	0.66	0.6 (-0.3, 1.6)	0.16	-0.4 (-1.8, 1.0)	0.58
Gait speed (m/sec)[a]	-0.02 (-0.06, 0.01)	0.11	-0.05 (-0.07, -0.03)	<0.001	0.03 (-0.01, 0.06)	0.19
**Males**	*n=179*		*n=264*			
PCS	4.4 (3.1, 5.7)	<0.001	0.7 (-0.3, 1.8)	0.18	3.7 (2.0, 5.4)	<0.001
MCS	-1.4 (-2.6, -0.2)	0.02	-0.2 (-1.1, 0.8)	0.72	-1.3 (-2.8, 0.3)	0.11
Physical function	4.2 (2.7, 5.8)	<0.001	1.0 (-0.2, 2.3)	0.10	3.2 (1.2, 5.2)	0.002
Role physical	1.1 (-0.3, 2.5)	0.11	-0.1 (-1.3, 1.0)	0.80	1.3 (-0.5, 3.1)	0.17
Bodily pain	6.0 (4.5, 7.5)	<0.001	0.5 (-0.7, 1.8)	0.39	5.5 (3.5, 7.4)	<0.001
General health	1.1 (-0.1, 2.3)	0.07	0.7 (-0.3, 1.6)	0.19	0.5 (-1.1, 2.0)	0.57
Vitality	0.6 (-0.8, 2.0)	0.42	-0.1 (-1.2, 1.0)	0.86	0.7 (-1.2, 2.5)	0.47
Social functioning	0.5 (-0.7, 1.8)	0.39	0.6 (-0.4, 1.6)	0.26	-0.04 (-1.6, 1.6)	0.97
Role emotional	0.3 (-1.0, 1.5)	0.69	-0.3 (-1.3, 0.7)	0.56	0.6 (-1.1, 2.2)	0.50
Mental health	-0.2 (-1.5, 1.1)	0.76	0.2 (-0.9, 1.3)	0.67	-0.4 (-2.2, 1.3)	0.62
Gait speed (m/sec)[b]	-0.01 (-0.04, 0.03)	0.77	-0.04 (-0.07, -0.02)	0.002	0.04 (-0.01, 0.08)	0.12

CI: confidence interval; PCS: Physical Component Summary score of SF-12; MCS: Mental Component Summary score of SF-12
All analyses adjusted for baseline body mass index, education, morbidities or chronic conditions, and days between outcome measures

[a]n=189 for female participants with hip replacement and n=327 for female controls
[b]n=139 for male participants with hip replacement and n=245 for male controls

Table S3. Baseline, follow-up, and changes in health status and gait speed in participants with hip replacement and age- and sex-matched controls excluding participants with self-reported joint replacement prior to ASPREE trial

	Participants with hip replacement N=274			Age- and sex-matched controls N=543			Difference between groups	
	Baseline (preoperative) Mean (SD)	Follow-up Mean (SD)	Change Mean (95% CI)¶	Baseline (study entry) Mean (SD)	Follow-up Mean (SD)	Change Mean (95% CI)¶	Change Mean (95% CI)¶	P¶
PCS	39.9 (11.1)	45.0 (10.5)	5.0 (3.9, 6.1)	48.6 (8.6)	48.5 (8.9)	0.1 (-0.6, 0.9)	4.9 (3.6, 6.2)	<0.001
MCS	56.7 (8.0)	55.6 (6.8)	-1.5 (-2.4, -0.5)	55.7 (7.2)	55.8 (6.7)	0.1 (-0.6, 0.7)	-1.5 (-2.7, -0.3)	0.01
Physical function	40.8 (11.1)	44.5 (11.4)	3.8 (2.6, 5.0)	48.0 (9.7)	48.1 (10.0)	0.3 (-0.6, 1.1)	3.6 (2.1, 5.0)	<0.001
Role physical	44.3 (9.8)	47.4 (9.4)	2.8 (1.7, 3.9)	49.9 (8.1)	50.0 (8.3)	0.3 (-0.5, 1.0)	2.6 (1.2, 3.9)	<0.001
Bodily pain	40.3 (10.8)	47.2 (10.7)	6.7 (5.3, 8.0)	50.6 (9.3)	50.5 (9.5)	0.002 (-0.9, 0.9)	6.7 (5.0, 8.3)	<0.001
General health	49.4 (9.1)	50.8 (9.0)	1.1 (0.1, 2.1)	52.3 (7.2)	52.1 (7.8)	-0.1 (-0.8, 0.6)	1.2 (-0.02, 2.4)	0.05
Vitality	51.6 (9.5)	52.8 (8.4)	1.0 (-0.1, 2.1)	55.1 (8.3)	54.7 (8.0)	-0.2 (-1.0, 0.6)	1.2 (-0.1, 2.5)	0.07
Social functioning	51.3 (9.2)	52.5 (7.7)	0.6 (-0.4, 1.6)	53.6 (7.1)	54.1 (6.3)	0.4 (-0.3, 1.1)	0.2 (-1.0, 1.4)	0.75
Role emotional	50.4 (8.7)	50.7 (7.7)	0.2 (-0.9, 1.2)	51.8 (6.7)	51.7 (7.5)	-0.1 (-0.9, 0.6)	0.3 (-1.0, 1.6)	0.66
Mental health	54.2 (7.9)	54.4 (7.2)	-0.1 (-1.1, 1.0)	55.0 (7.9)	55.2 (7.7)	0.3 (-0.4, 1.1)	-0.4 (-1.7, 0.9)	0.57
Gait speed (m/sec)*	0.95 (0.24)	0.94 (0.21)	-0.01 (-0.04, 0.02)	1.06 (0.22)	1.01 (0.22)	-0.05 (-0.07, -0.03)	0.04 (-0.001, 0.07)	0.05

SD: standard deviation; CI: confidence interval; PCS: Physical Component Summary score of SF-12; MCS: Mental Component Summary score of SF-12

*n=197 for participants with hip replacement and n=495 for controls

¶Adjusted for baseline body mass index, education, morbidities or chronic conditions, and days between outcome measures
